# Supplementary material for: Altered expression of maize PLASTOCHRON1 enhances biomass and seed yield by extending cell division duration
Source: Nat Commun. 2017 Mar 16;8:14752. doi: 10.1038/ncomms14752 (PMC5356070; doi:10.1038/ncomms14752)
Supplement: Supplementary Information — Supplementary Figures and Supplementary Tables [file ncomms14752-s1.pdf]

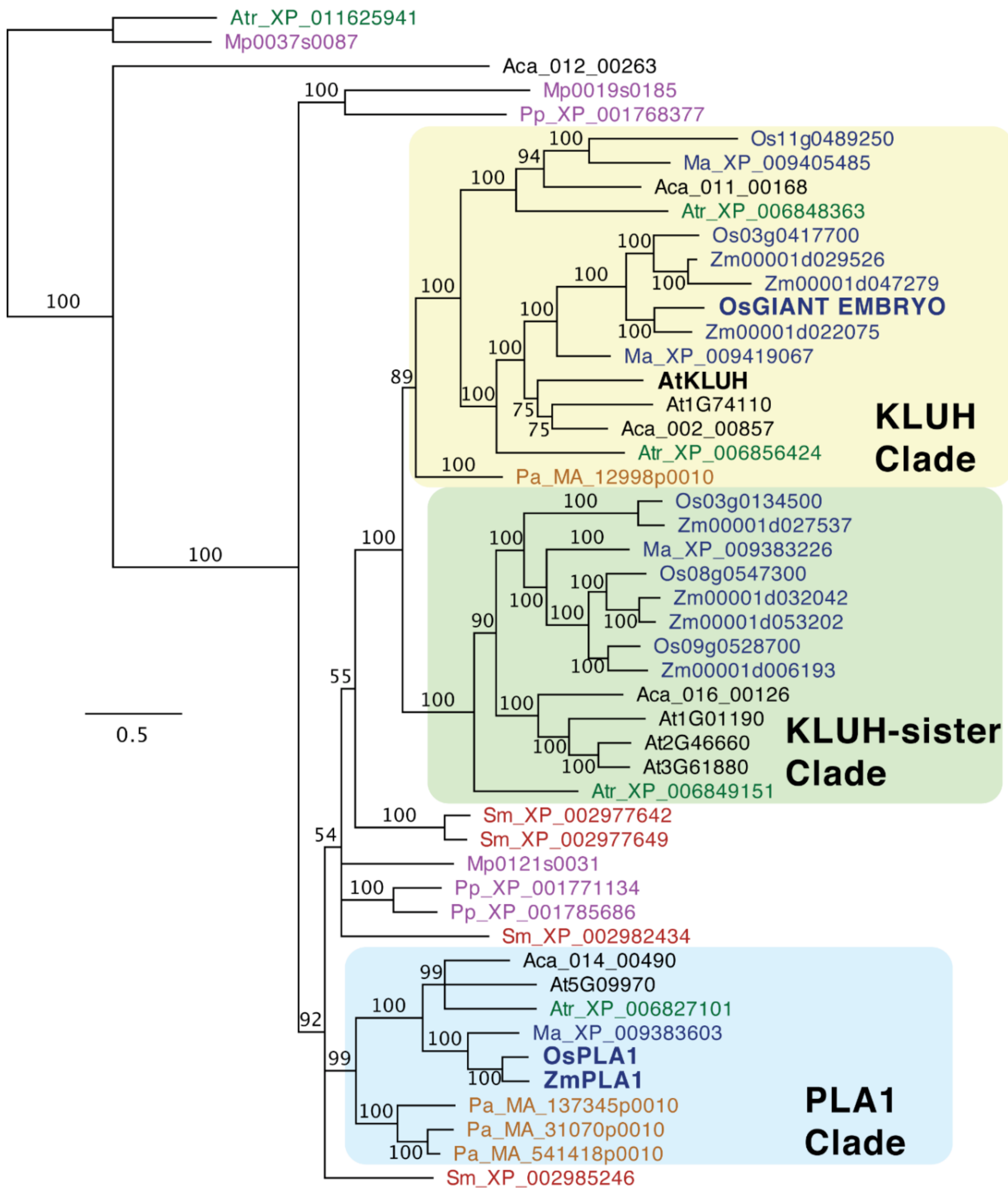

Supplemental Figure 1. Land plant CYP78A phylogeny

Majority rule consensus tree from a Bayesian analysis of CYP78A predicted protein sequences from land plants including representative taxa from eudicots (*Arabidopsis thaliana*-At, and *Aquilegia caerulia*-Aca: black), monocots (*Zea mays*-Zm, *Oryza sativa*-Os, and *Musa acuminata*-Ma: blue), basal angiosperm (*Amborella trichopoda*-Atr: green), gymnosperm (*Picea abies*-Pa: orange), lycopod (*Selaginella moellendorffii*-Sm: red), and bryophytes (*Physcomitrella patens*-Ppa, and *Marchantia polymorpha*-Mp: purple). Three well-supported clades (KLUH, KLUH-sister and PLA1) appear to have arisen in a common ancestor of all seed plants (gymnosperms and angiosperms). While the sister relationship of KLUH and KLUH-sister clades is well-supported, the timing of the gene duplication that gave rise to these lineages and the PLA1 clade is not clear due to poor resolution of outgroups to these clades. ZmPLA1 is confidently identified as the sole maize ortholog of the rice PLASTOCHRON1 (PLA1) gene. Node support is indicated as Bayesian posterior probability.

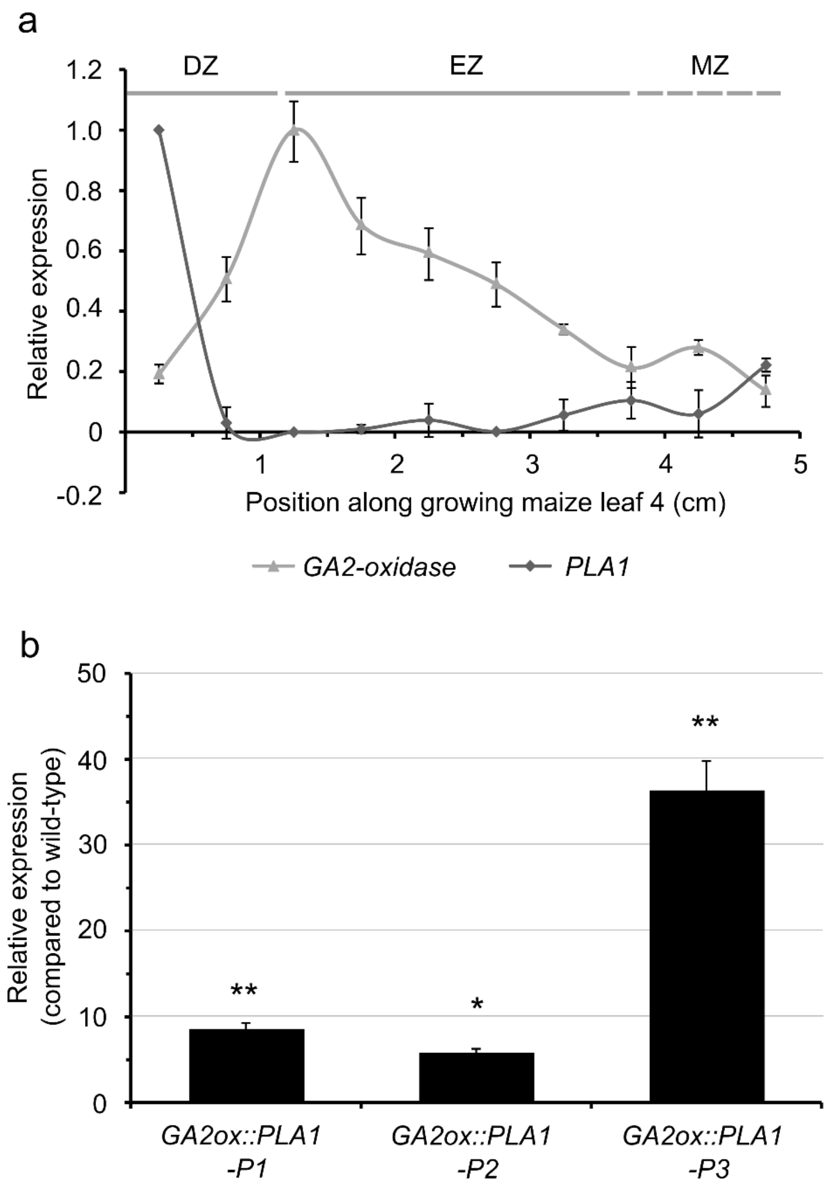

Supplementary Figure 2. *PLA1* expression level in wild type and two *PLA1* overexpression lines. (**a**) *PLA1* and *GA2-oxidase* expression along maize leaf four in B104 (wild-type) relative to 18S. The dotted line indicates the mature zone that continues after centimeter 5. (**b**) Relative expression of *PLA1* at the basal 0.5-cm region of the fourth leaf of three *GA2ox:PLA1* transgenic backcrossed lines, compared with their corresponding leaf samples in non-transgenic siblings. Error bars indicate standard errors. * and ** indicate significant differences with p-values < 0.05 and < 0.001, Student's t-test, n=3).

DZ: division zone; EZ: expansion zone; MZ: mature zone.

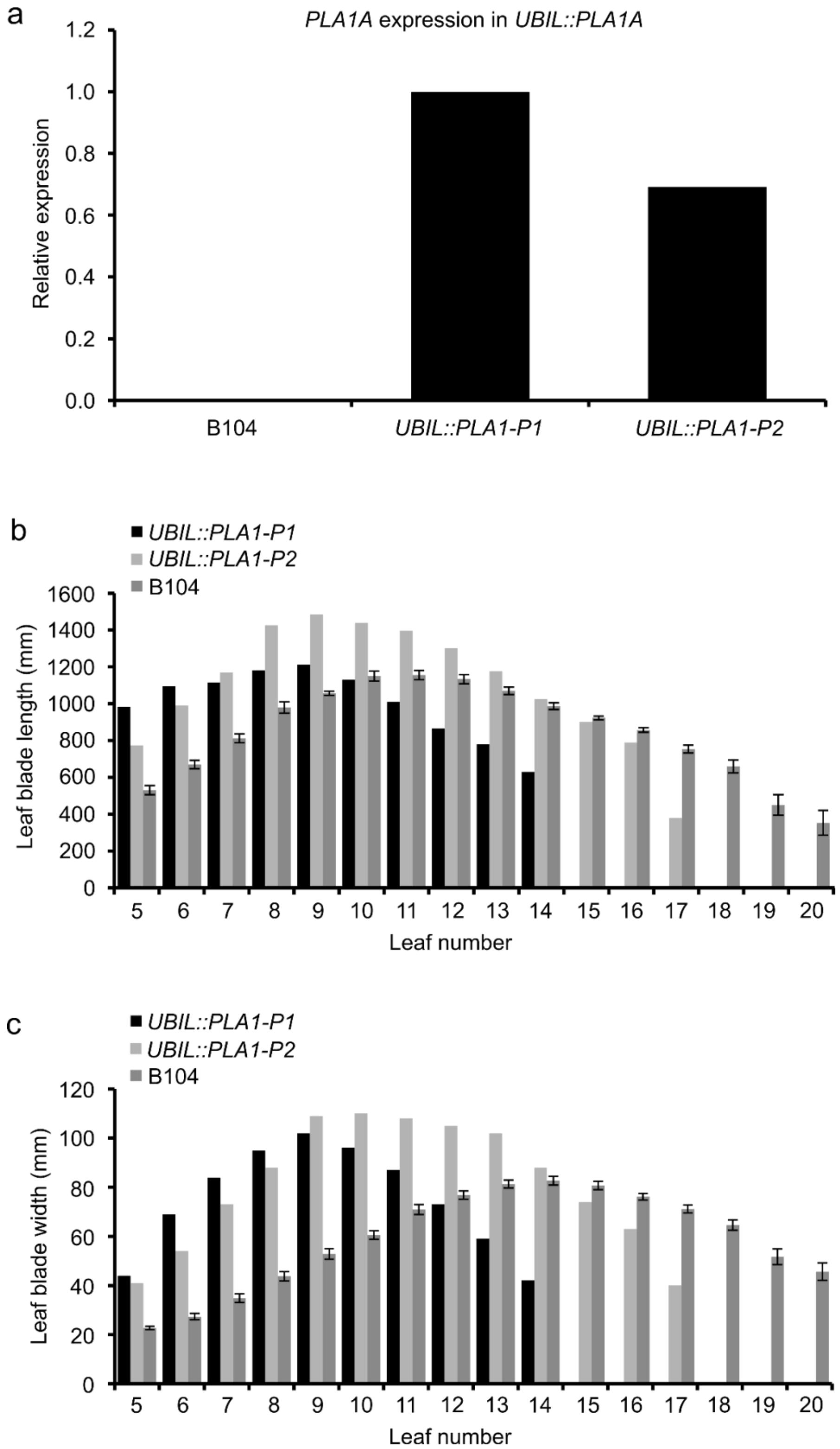

Supplementary Figure 3. Leaf phenotype in two *PLA1* overexpression lines *UBIL:PLA1-P1* and *UBIL:PLA1-P2*. (**a**) Relative *PLA1* expression levels for B104 compared with *UBIL:PLA1-P1* and *UBIL:PLA1-P2* in mature samples of the eighth leaf. (**b-c**) Final leaf length and width of the two *UBIL:PLA1* plants compared with the average of eleven B104 plants. Error bars indicate standard errors (n=11).

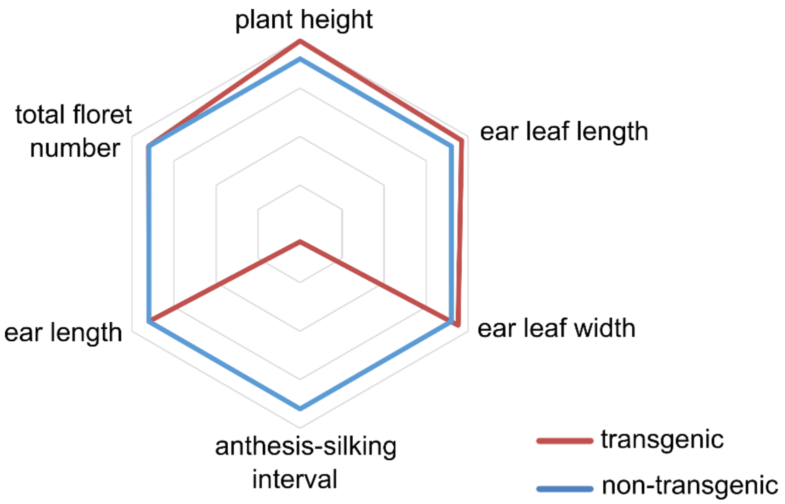

Supplementary Figure 4. Phenotypic evaluations of the *GA2ox:PLA1-P2* transgenic lines in the U.S. field trial in 2015. The scale of the spider web is from -80% to 120% (n=16-23). Data are presented as measurements from plants containing the *GA2ox:PLA1* construct relative to the non-transgenic controls.

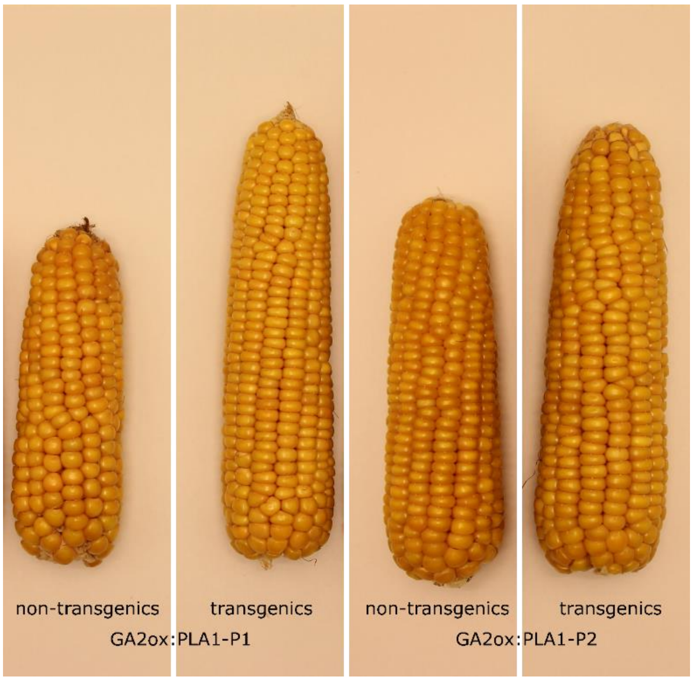

Supplementary Figure 5. Pictures of representative cobs of transgenic (T) and non-transgenic (NT) plants of *GA2ox:KLUH-P1*xCML91 and *GA2ox:KLUH-P2*xCML91 grown in the field in 2015 in Belgium. Scale bar = 5 cm.

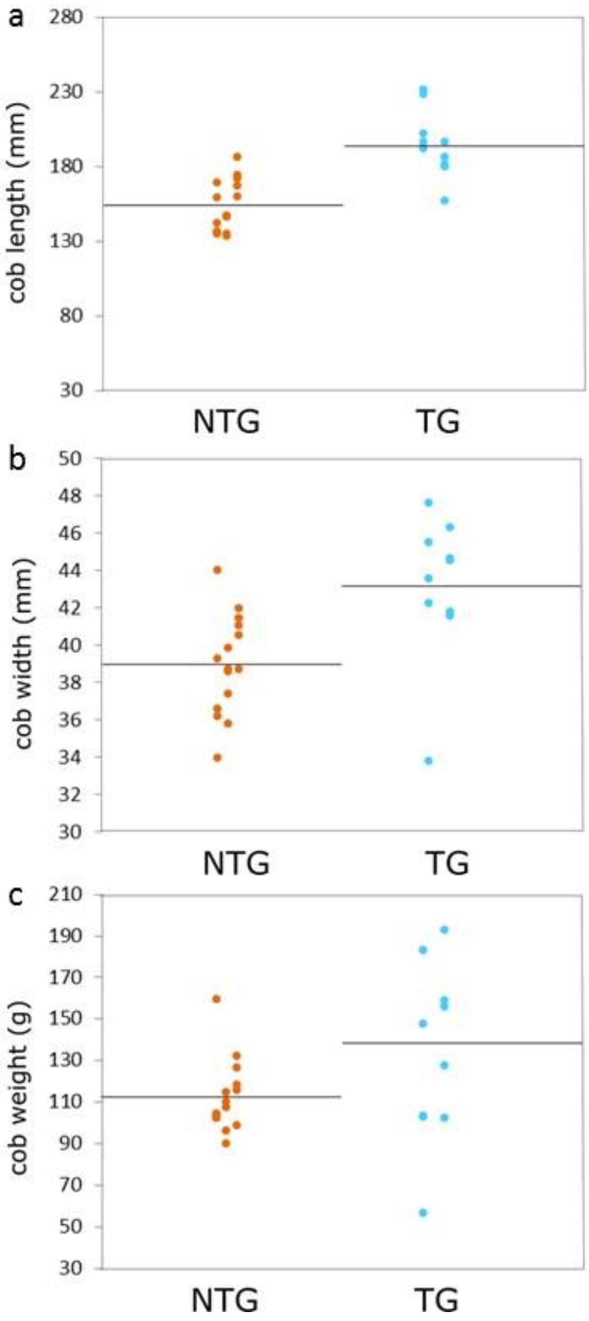

Supplementary Figure 6. Variation of the seed yield parameters cob length (a), width (b) and weight (c) in *GA2OX:PLA1 line 2* in the 2015 field trial in Belgium. The dots represent the individual data points and the line the average. The cob length, width and weight were significantly different between the transgenic (TG) and non-transgenic (NTG) plants (p- value ≤ 0.01), according to the Student's t-test.

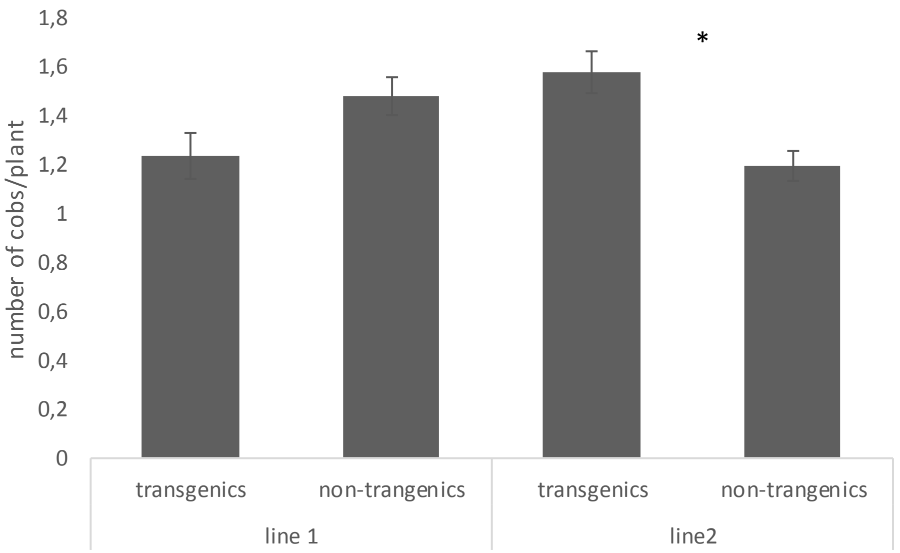

Supplementary Figure 7. Number of cobs per plant in the 2015 field trial in Belgium for the *GA2OX:PLA1* transgenic and non-transgenic plants. Error bars represent standard error. The data were collected as averages of three randomized blocks per genotype. Each block consisted of four rows with a minimum of thirty two plants per row. The asterisk indicates significant differences (p-value < 0.01, Student's t-test, n=3).

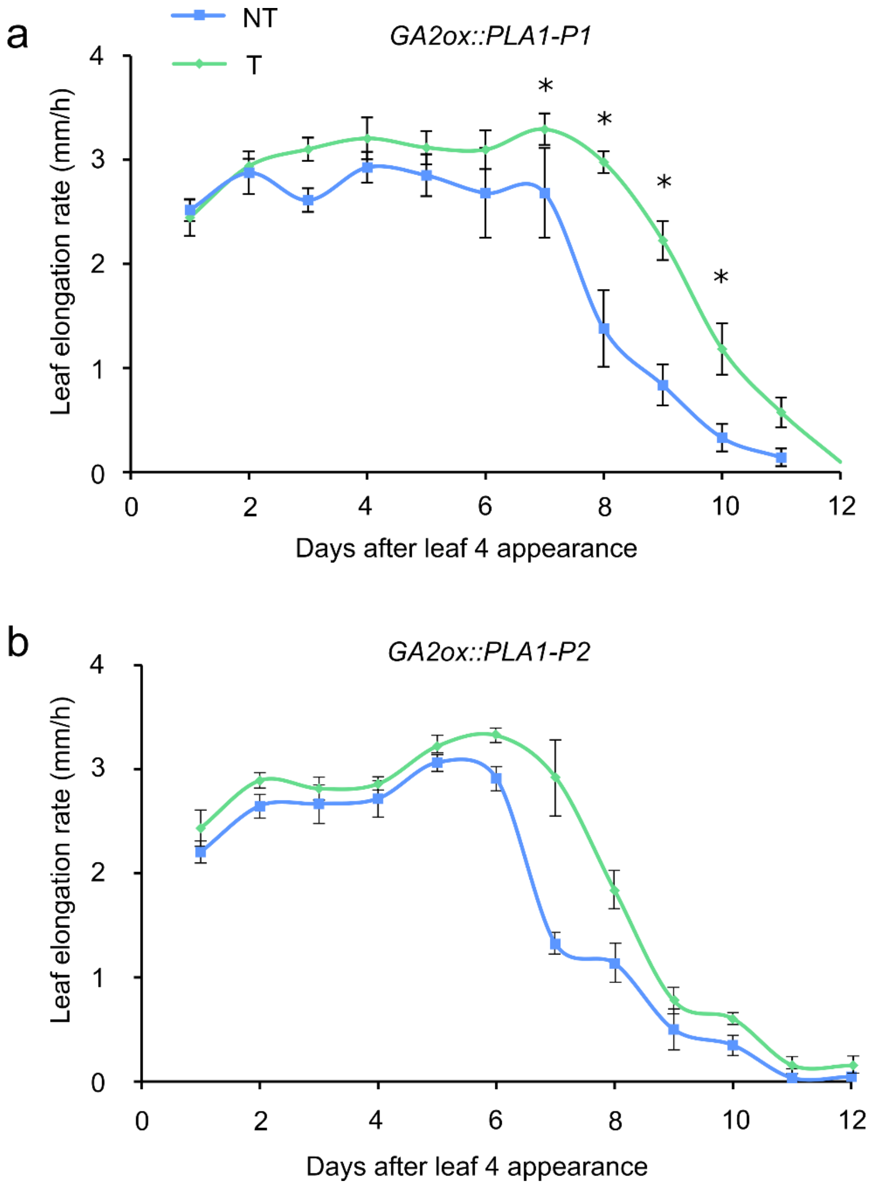

Supplementary Figure 8. Leaf elongation rate of leaf 4 in (**a**) *GA2ox:PLA1-P1* and (**b**) *GA2OX:PLA1-P2* relative to the non-transgenic siblings. * indicates significant differences between the non-transgenics and the transgenics ($p < 0.05$, mixed model analysis with custom hypothesis Wald tests (corrected for multiple testing, n=5).

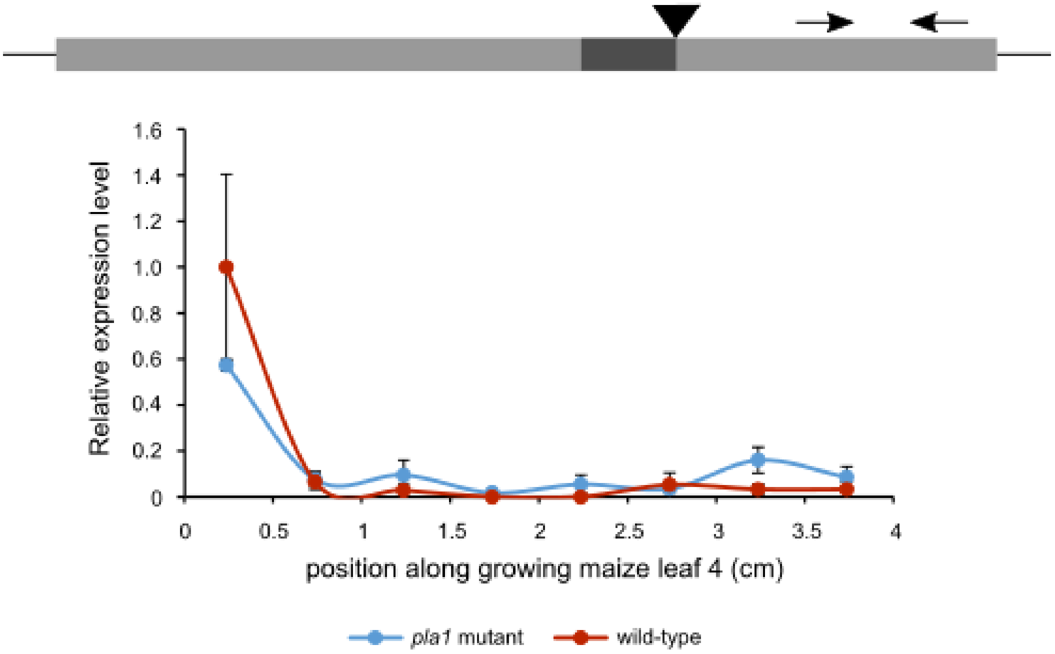

Supplementary Figure 9. The *pla1* mutant. Overview of the gene structure, transposon insertion site and primers for Q-PCR. Gray boxes indicate exons; black box the intron; the triangle represents the transposon insertion site; the arrows represent the RT-qPCR primers. Relative expression of *PLA1* in the mutant compared with the wild-type. Expression levels were normalized relative to 18S rRNA. Error bars represent standard errors (n = 3 pools of three plants).

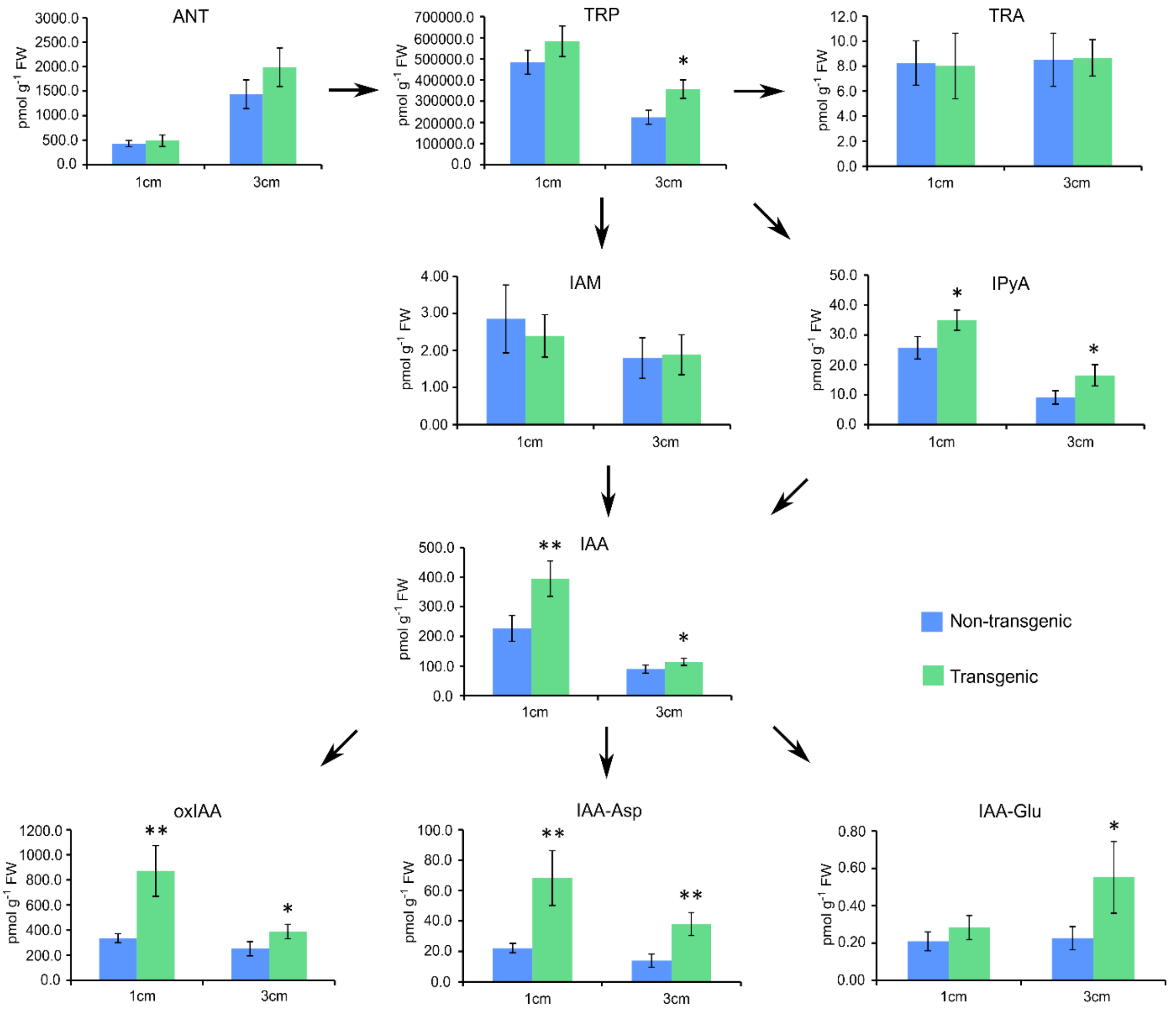

Supplementary Figure 10. Endogenous auxin content in *GA2ox:PLA1-P3* transgenic plants. Two segments of the growth zone of the fourth leaf (0.5-1 cm and 2.5-3 cm) of transgenic plants and non-transgenic siblings were harvested at the second day after leaf appearance. Error bars indicate standard errors (n = 5). * and ** indicate significant differences between genotypes with p-values < 0.05 and < 0.001, respectively, mixed model analysis with custom hypothesis Wald tests (corrected for multiple testing) (n=3). ANT, anthranilate; TRP, tryptophan; TRA, tryptamine; IAM, indole-3-acetamide; IPyA, indole-3-pyruvic acid; IAA, indole-3-acetic acid; oxIAA, 2-oxoindole-3-acetic acid; IAA-Asp, IAA-aspartate; IAA-Glu, IAA-glutamate.

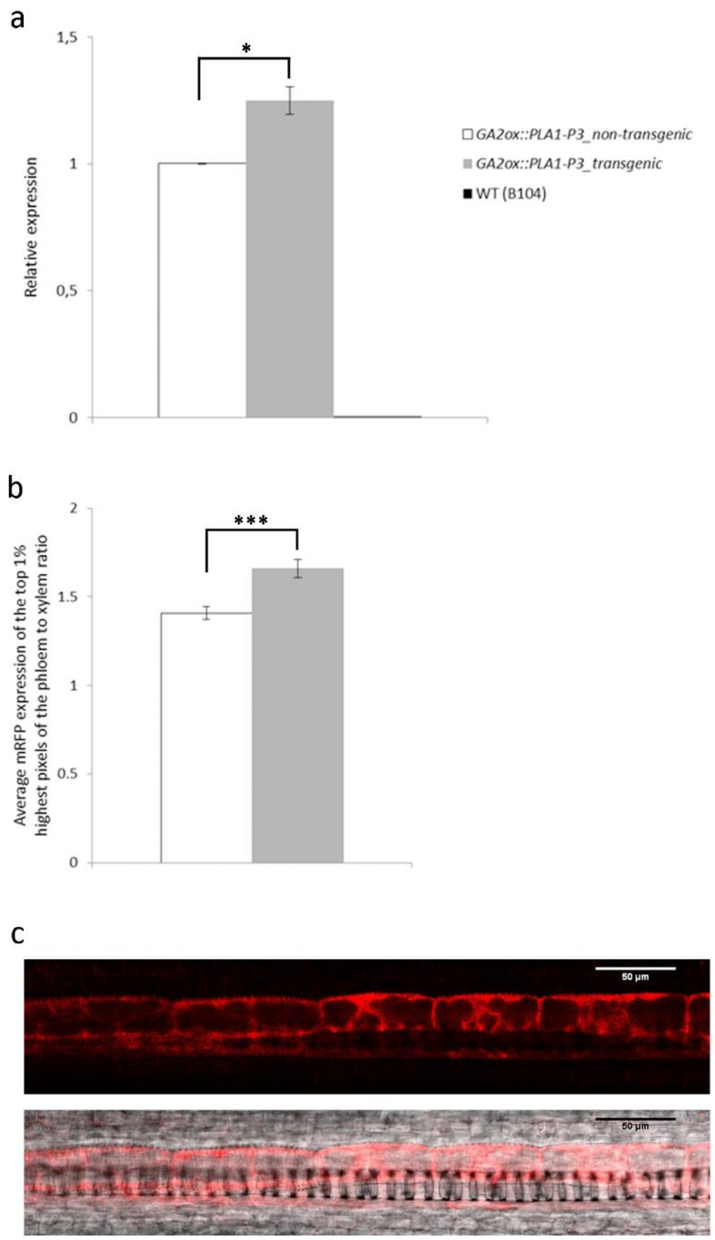

Supplemental figure 11. DR5rev:mRFPer marker introgressed in *GA2ox:PLA1-P3* transgenic and non-transgenic plants in the most basal half cm of leaf four, six days after emergence. (a) mRFP marker expression in the most basal half cm of leaf four, six days after emergence, in *GA2ox:PLA1-P3* transgenic plants compared to *GA2ox:PLA1-P3* non-transgenic plants, determined by RT-qPCR. Wild type (WT) B104 plants were taken as a negative control. The error bars show standard error of the mean (SEM) of three biological replicates (two technical repeats each). *p < 0.03, Student's t-test, n=3. (b) Quantification of *DR5rev:mRFPer* expression in the most basal half cm of leaf four, using confocal images, and presented as average mRFP intensity of the top 1% highest pixels of the phloem to xylem ratio in *GA2ox:PLA1-P3* transgenic plants compared to *GA2ox:PLA1-P3* non-transgenic plants. The error bars show standard error of the mean (SEM). t-test, *** p < 0.0001, Student's t-test, n = 9 leaves per line. (c) mRFP (*DR5rev:mRFPer*) localization in the ER of phloem cells surrounding the xylem in the most basal half cm of leaf four, two days after emergence, in a *GA2ox:PLA1-P3* non-transgenic plant, without and with the transmitted light channel. Scale bars: 50µm.

Supplementary Table 1. Percentage difference of the *GA2ox:PLA1* transgenic hybrids compared with the non-transgenic hybrids.

	CML91	F7	H99	Mo17	W153R
**FLL**	12.75 *	9.90 **	12.62 *	12.44 **	12.48 **
**WIDTH**	7.03	15.73 **	13.82 *	7.93 *	13.43 *
**AREA**	24.06 *	29.41 **	33.27 **	20.52 **	48.74 **

Statistics were calculated based on two-tailed Student's t-test. * and ** indicate significant differences with $p \leq 0.05$ and 0.01, respectively, Students's t-test, n=4-19. FLL, final leaf length.

Supplementary Table 2. Kinematic analysis of *GA2ox:PLA1-P1* and *GA2ox:PLA1-P2* transgenic and non-transgenic siblings.

Growth parameters [a]	*GA2ox:PLA1-P1*				*GA2ox:PLA1-P2*			
	NT	T	p-value [b]	Δ%	NT	T	p-value [b]	Δ%
Final leaf length (mm)	**585.38**	**705.14**	**0.00**	**20.46**	**572.27**	**680.92**	**0.01**	**18.98**
LERmax (mm h^{-1})	2.74	3.08	0.07	12.40	2.63	2.81	0.43	6.85
LED (h)	**232.3**	**264.17**	**0.00**	**13.71**	**235.26**	**264.17**	**0.02**	**12.28**
Division zone size (cm)	1.26	1.29	0.82	2.26	1.23	1.31	0.60	6.16
Dividing cell size (µm)	17.41	17.07	0.52	-1.93	17.76	18.09	0.85	1.84
Number dividing cells	737.77	757.75	0.79	2.71	709.41	739.04	0.64	4.18
Cell production (Cells h^{-1})	21.58	21.46	0.83	-0.58	20.09	21.08	0.17	4.92
Cell division (Cells $cell^{-1}h^{-1}$)	0.03	0.03	0.70	-4.04	0.03	0.03	0.92	0.88
Cell cycle duration (h)	23.76	24.45	0.80	2.87	24.51	24.30	0.92	-0.84
Mature cell size (µm)	**109.67**	**120.61**	**0.02**	**9.98**	**105.69**	**114.82**	**0.03**	**8.63**
Elongation zone size (cm)	2.53	2.72	0.64	7.37	2.59	2.96	0.33	14.23
Elongating cell size (µm)	62.76	64.82	0.78	3.28	59.39	68.62	0.20	15.55
Number elongating cells	406.77	417.89	0.81	2.73	436.15	432.18	0.93	-0.91

[a] Mean value for each parameter was determined for three plants, except final leaf length and leaf elongation rate that were determined using five plants. All parameters were determined at 48 h after leaf emergence from the sheath.

[b] Statistical significance based on Student's t-test. Numbers in bold indicate parameters that are statistically different ($p \leq 0.05$), n=3.

LERmax: maximal leaf elongation rate; LED: leaf elongation duration. NT: non-transgenics. T: transgenics.

Supplementary Table 3. Leaf four phenotype of transgenic plants expressing both *GA2ox:PLA1* and *UBIL:GA20ox-1,* relative to the single overexpression plants and the null segregants, grown in the growth chamber.

	length (mm)	width (mm)	area (mm^2)
UBIL:GA20ox-1_T	770,5±4	17,3±2	8965,0±3
UBIL:GA20ox-1_NT	565,2±7	20,4±1	8634,0±2
GA2ox:PLA1_T	707,7±2	25,4±3	10866,7±4
GA2ox:PLA1_NT	548,3±9	22,2±1	8287,5±8
UBIL:GA20ox-1_T×GA2ox:PLA1_T	912,3±8	24,2±1	13216,7±2
UBIL:GA20ox-1_NT×GA2ox:PLA1_NT	608,5±6	19±2	8876,7±2

The cross of *GA2ox:PLA1* and *UBIL:GA20ox-1* was made by crossing segregating *GA2ox:PLA1* with segregating *UBIL:GA20ox-1*.

T: transgenics; NT: non-transgenics.

Average ± SE; n=3-5.

Supplementary Table 4. Leaf growth parameters of *GA2ox:PLA1* segregating lines under mild drought (a) and *GA2ox:PLA1-P3* segregating line under cold night (b) conditions.

a		control				mild drought			
		Transgenic	non-transgenic	Δ%	p-value	Transgenic	non-transgenic	Δ%	p-value
GA2ox:PLA1-P1	FLL (mm)	703.4±8.71	540.3±10.98	30.2	6.00E-09	589.9±25.36	491.8±24.1	23.6	0.02
	LERmax (mm/h)	3.4±0.07	3.1±0.09	10.1	0.007	2.2±0.11	2.2±0.24	1.5	0.8
	LED (h)	237.5±5.57	193.4±5.0	22.8	9.00E-06	293.2±15.86	219.2±13.9	33.8	0.004
GA2ox:PLA1-P2	FLL (mm)	643.8±8.68	557.5±15.85	14.7	5.00E-04	573.8±11.46	478.6±13.55	21.6	1.00E-04
	LERmax (mm/h)	3.4±0.07	3.1±0.12	9.4	0.05	2.4±0.16	2.2±0.13	8.7	0.6
	LED (h)	209.4±3.6	191.6±4.48	9.3	0.006	268.5±18.09	217.6±6.16	23.4	0.04
GA2ox:PLA1-P3	FLL (mm)	677.7±7.75	536.3±8.34	26.5	2.00E-09	560.5±14.9	487.4±12.85	19.8	4.00E-04
	LERmax (mm/h)	3.3±0.08	3.0±0.07	10.8	0.01	2.2±0.13	2.2±0.11	-3	0.7
	LED (h)	230.6±7.95	187.9±2.46	22.7	0.002	297.0±8.26	222.2±6.34	33.7	2.00E-05

b		control				cold night			
		Transgenic	non-transgenic	Δ%	p-value	transgenic	non-transgenic	Δ%	p-value
GA2ox:PLA1-P3	FLL (mm)	791.8±11.9	634.9±10.8	24.7	3.00E-09	671.8±7.5	513.08±10.8	30.9	1.00E-10
	LERmax (mm/h)	3.38±0.04	3.04±0.03	11.4	4.00E-07	2.5±0.04	2.24±0.05	11	6.00E-04
	LED (h)	271.0±4.2	227.9±3.5	18.8	1.00E-07	299.5±4.5	233.5±4.5	28	2.00E-10

Average±SE. Statistical significance based on Student's t-test, n=5-15.

FLL: final leaf length (mm); LERmax: maximal leaf elongation rate; LED: leaf elongation duration.

Supplementary Table 5. Enrichment analysis of gene ontology (GO) terms for differentially expressed transcriptions in *GA2ox:PLA1-P3* segregating plants during different developmental stages. GO categories that are in common in the comparison between the two time points (during and after steady state growth) in both the non-transgenic and transgenic plants are highlighted in red and blue for the upregulated and downregulated processes, respectively.

A	genes upregulated in WT at day6 compared with WT at day2	genes downregulated in WT at day6 compared with WT at day2
	membrane lipid catabolic process	negative regulation of cyclin-dependent protein serine/threonine kinase activity
	photosynthesis	positive regulation of phytoalexin biosynthetic process
	sulfate transport	cell cycle arrest
	salicylic acid metabolic process	cytokinin biosynthetic process
	cellulose metabolic process	regulation of systemic acquired resistance
	secondary cell wall biogenesis	auxin metabolic process
	ATP hydrolysis coupled proton transport	maintenance of meristem identity
	response to light	hormone biosynthetic process
	photosynthetic electron transport chain	stem cell maintenance
	anion transport	nucleobase metabolic process
	auxin mediated signaling pathway	glutamine family amino acid biosynthetic process
	response to karrikin	phyllome development
	organic acid transport	shoot system development
	carboxylic acid transport	regulation of cellular biosynthetic process
	lipid localization	regulation of nitrogen compound metabolic process
	response to cold	response to endogenous stimulus
	response to oxidative stress	regulation of macromolecule metabolic process
	response to organic substance	cellular response to stress
	single-organism transport	heterocycle biosynthetic process
	lipid metabolic process	

B	genes upregulated in *GA2ox:PLA1* at day6 compared with *GA2ox:PLA1* at day2	genes downregulated in *GA2ox:PLA1* at day6 compared with *GA2ox:PLA1* at day2
	photosynthesis	regulation of phytoalexin biosynthetic process
	sulfate transport	cotyledon vascular tissue pattern formation
	response to light	detection of fungus
	auxin mediated signaling pathway	benzoate metabolic process
	response to auxin	cellular response to high light intensity
	lipid localization	syncytium formation
	organic acid transport	cell cycle arrest
	carboxylic acid transport	regulation of systemic acquired resistance
	response to cold	primary root development
	cellular glucan metabolic process	abscisic acid metabolic process
	glucan metabolic process	apocarotenoid metabolic process

	response to abiotic stimulus	auxin metabolic process
	lipid metabolic process	maintenance of meristem identity
	transmembrane transport	cellular carbohydrate catabolic process
	carbohydrate metabolic process	cellular response to gibberellin stimulus
	single-organism transport	hormone biosynthetic process
		disaccharide biosynthetic process
		negative regulation of developmental process
		response to auxin
		shoot system development
		regulation of cellular biosynthetic process
		regulation of nitrogen compound metabolic process

**C**	genes upregulated in *GA2ox:PLA1* at day2 compared with WT at day2	genes downregulated in *GA2ox:PLA1* at day2 compared with WT at day2
	lactate transport	aromatic amino acid transport
	oxidation-reduction process	floral organ development
		perianth development
		pyrimidine nucleobase salvage
		positive regulation of epidermis development
		positive regulation of stomatal complex development
		regulation of growth
		pattern specification process

**D**	genes upregulated in *GA2ox:PLA1* at day6 compared with WT at day6	genes downregulated in *GA2ox:PLA1* at day6 compared with WT at day6
	negative regulation of DNA binding transcription factor activity	terpenoid catabolic process
	regulation of hormone levels	auxin polar transport
	regulation of transcription. DNA-dependent	response to karrikin
		lipid transport
		fatty acid biosynthetic process

(a-b) GO enrichment of the 2,444 and 1,845 transcripts differentially expressed in non-transgenic (a) and *GA2ox:PLA1-P3* (b) leaf samples over time, respectively; (C-D) GO enrichment of the 115 and 533 differentially expressed transcripts in *GA2ox:PLA1-P3* compared with the non-transgenic siblings at day two (C) and day six (D) after emergence of leaf four, respectively.

GO enrichment is characterized by PLAZA[52] with a P-value<0.01 as cut-off, and is listed from the highest enriched to less enriched categories.

Supplementary Table 6. Expression level of auxin efflux transporters in *GA2ox:PLA1-P3* segregating plants during different developmental stages.

The expression level was calculated by log fold change (logFC) and indicated the change for the front samples compared with the next. Purple: logFC>1; blue: logFC<-1.

	geneID	*GA2ox:PLA1* at day2 vs WT at day2	*GA2ox:PLA1* at day6 vs WT at day6	*GA2ox:PLA1* at day6 vs *GA2ox:PLA1* at day2	WT at day6 vs WT at day2
LAX3	GRMZM2G149481			-1.04	
ZmPIN1a	GRMZM2G098643			-1.48	-1.28
ZmPIN1b	GRMZM2G074267		-1.17	1.2	1.87
PIN1c	GRMZM2G149184			4.77	4.93
PIN10a	GRMZM2G126260		-1.52	1.55	2.93
PIN1d	GRMZM2G171702				-1.15
ZmPINY	GRMZM2G050089			1.12	1.43
BIF2/positive regulator of cellular auxin efflux	GRMZM2G103559		-1.12	1.29	2.34
BIF2/positive regulator of cellular auxin efflux	GRMZM2G177812				1.06

Supplementary Table 7. Primers used to clone fragments, to genotype and to check the expression level.

goal	amplicon	template > fragment	primer sequences 5' to 3'
cloning	PLA1_cds	cDNA > fragment A	ATGGCGATGGCCTCCGCGGCTTGCTCATG
			TTATATGCATGTACAGTATGTTTTTTTCAG
		cDNA > fragment B	CTGAAAAAAACATACTGTACATGCATATAA
			TCAGGCGGTGCGGGGGATGG
		fragment A+B > PLA1A CDS	GGGGACAAGTTTGTACAAAAAAGCAGGCTCCATGGCGATGGCC TCCGCGGCTTGCTCATG
			GGGGACCACTTTGTACAAGAAAGCTGGGTCTCAGGCGGTGCGG GGGATGG
	GA2OX promoter	gDNA	GGGGACAACTTTGTATAGAAAAGTTGCCGAGGATTGCAGCTCCT GGATC
			GGGGACTGCTTTTTTGTACAAACTTGCGGCTGTCTGGCCGTGC GGATG
expression level	PLA1	cDNA	CTCTGCAACGGCATGGTGGTC
			GGCGGCATCGTGGGTTATGG
	18S rRNA	cDNA	ACCTTACCAGCCCTTGACATATG
			GACTTGACCAAACATCTCACGAC
genotyping	UBI::PLA1	gDNA	TACGCTATTTATTTGCTTGG
			ATCGACGTCGATGCTC
	GA2OX::PLA1	gDNA	AGTTATTGATGAGCCGAGTC
			GAACGCCATGAGCTCCTTGG
	mRFP		CCTCAGTTCCAGTACGGCTC
			TGTAGATGAACTCGCCGTCC
	BASTA	gDNA	ACCTTACCAGCCCTTGACA
			GACTTGACCAAACATCTCA
